# Early Jurassic dinosaur fetal dental development and its significance for the evolution of sauropod dentition

**DOI:** 10.1038/s41467-020-16045-7

**Published:** 2020-05-07

**Authors:** Robert R. Reisz, Aaron R. H. LeBlanc, Hillary C. Maddin, Thomas W. Dudgeon, Diane Scott, Timothy Huang, Jun Chen, Chuan-Mu Chen, Shiming Zhong

**Affiliations:** 1 Dinosaur Evolution Research Centre and International Centre of Future Science, Jilin University, Changchun, 130000 Jilin China; 2 Department of Biology, University of Toronto Mississauga, Mississauga, ON L5L 1C6 Canada; 3 National Chung Hsing University, Taichung, 40227 Taiwan; 4 Department of Biological Sciences, University of Alberta, Edmonton, AB T6G 2E9 Canada; 5 Department of Earth Sciences, Carleton University, Ottawa, ON K1S 5B6 Canada; 6 Chuxiong Prefectural Museum, Chuxiong, 675000 Yunnan China

**Keywords:** Evolutionary developmental biology, Palaeontology

## Abstract

Rare occurrences of dinosaurian embryos are punctuated by even rarer preservation of their development. Here we report on dental development in multiple embryos of the Early Jurassic *Lufengosauru*s from China, and compare these to patterns in a hatchling and adults. Histology and CT data show that dental formation and development occurred early in ontogeny, with several cycles of tooth development without root resorption occurring within a common crypt prior to hatching. This differs from the condition in hatchling and adult teeth of *Lufengosaurus*, and is reminiscent of the complex dentitions of some adult sauropods, suggesting that their derived dental systems likely evolved through paedomorphosis. Ontogenetic changes in successive generations of embryonic teeth of *Lufengosaurus* suggest that the pencil-like teeth in many sauropods also evolved via paedomorphosis, providing a mechanism for the convergent evolution of small, structurally simple teeth in giant diplodocoids and titanosaurids. Therefore, such developmental perturbations, more commonly associated with small vertebrates, were likely also essential events in sauropod evolution.

## Abstract (web summary)

Dinosaurs had some of the most complex dentitions known. Here, Reisz et al. characterize dental development across embryonic, hatchling and adult *Lufengosaurus*, an Early Jurassic sauropodomorph dinosaur, and suggest that derived sauropod dentition evolved by paedomorphosis (juvenilization).

## Introduction

Studying dinosaur tooth development provides crucial insights into dinosaur feeding behavior and evolution^1–4^. This is especially applicable to their earliest ontogenetic stages, because these reveal important information not only about patterns of tooth formation, but also aspects of reproductive behavior, and even parental care^5^. To date, embryonic teeth have been found sporadically in Late Jurassic and Late Cretaceous theropods^6–8^, Late Cretaceous titanosaurid sauropods^9,10^, and in a lambeosaurine ornithischian^11^. The anatomy and histology of embryonic teeth of derived sauropodomorph and ornithischian dinosaurs have been studied^10,11^, but we currently lack a good understanding of dinosaur tooth development at the earliest ontogenetic stages in other groups. Patterns of tooth development at the initial stages of ontogeny in some of the earliest known dinosaurs are particularly important for reconstructing the evolutionary history of teeth in a clade of tetrapods that demonstrates extreme dental variation, and includes some of the most complex dentitions in history^12,13^.

The famous fossil locality near the Dawa village in Lufeng County, Yunnan Province, China is well known for the preservation of numerous subadult and adult skeletons of *Lufengosaurus*, as well as a near complete skull and scattered postcranial material of a hatchling^14–16^. A more recently discovered bonebed^17^ in this classic locality has yielded more than 200 small skeletal elements, including cranial and dentigerous elements. The remains were previously identified as embryos based on the absence of erupted teeth in the preserved maxillae and dentaries, centra with open notochordal canals, and the extreme vascularization of the cortical bone tissues^17^. Although uncertain, comparisons with other in ovo embryonic dinosaur materials suggested that they represent the second half of fetal ontogeny^6^. They were also identified as pertaining to the sauropodomorph *Lufengosaurus*, based on a phylogenetic analysis and comparisons with the known sauropodomorphs from Yunnan Province^17^. In contrast to other embryonic dinosaur remains, usually found in situ within eggs and more or less articulated, these elements were preserved mainly isolated and amenable to detailed study in various views, using multiple methods.

For the present study, previously reported^17^ and new jaw elements were mechanically prepared and analyzed through a combination of high-resolution micro-computed tomography (HRµCT) and histological thin sections. These methods allow us to provide detailed evidence of embryonic dental development in this early sauropodomorph dinosaur. In order to contextualize these findings we compare the developmental data from the *Lufengosaurus* embryos with those in hatchling and adult individuals, and more broadly with available dental development and replacement data in other adult sauropodomorphs, including giant sauropods. We also compare the embryonic data from *Lufengosaurus* to dental developmental data from alligators as extant archosaurian models. Finally, we provide evidence of dramatic ontogenetic variation in dental development and replacement in *Lufengosaurus*, and discuss its significance for the evolution of sauropod dentitions^1,18^.

## Results

### Embryonic maxillary and dentary teeth

**Fig. 1:**
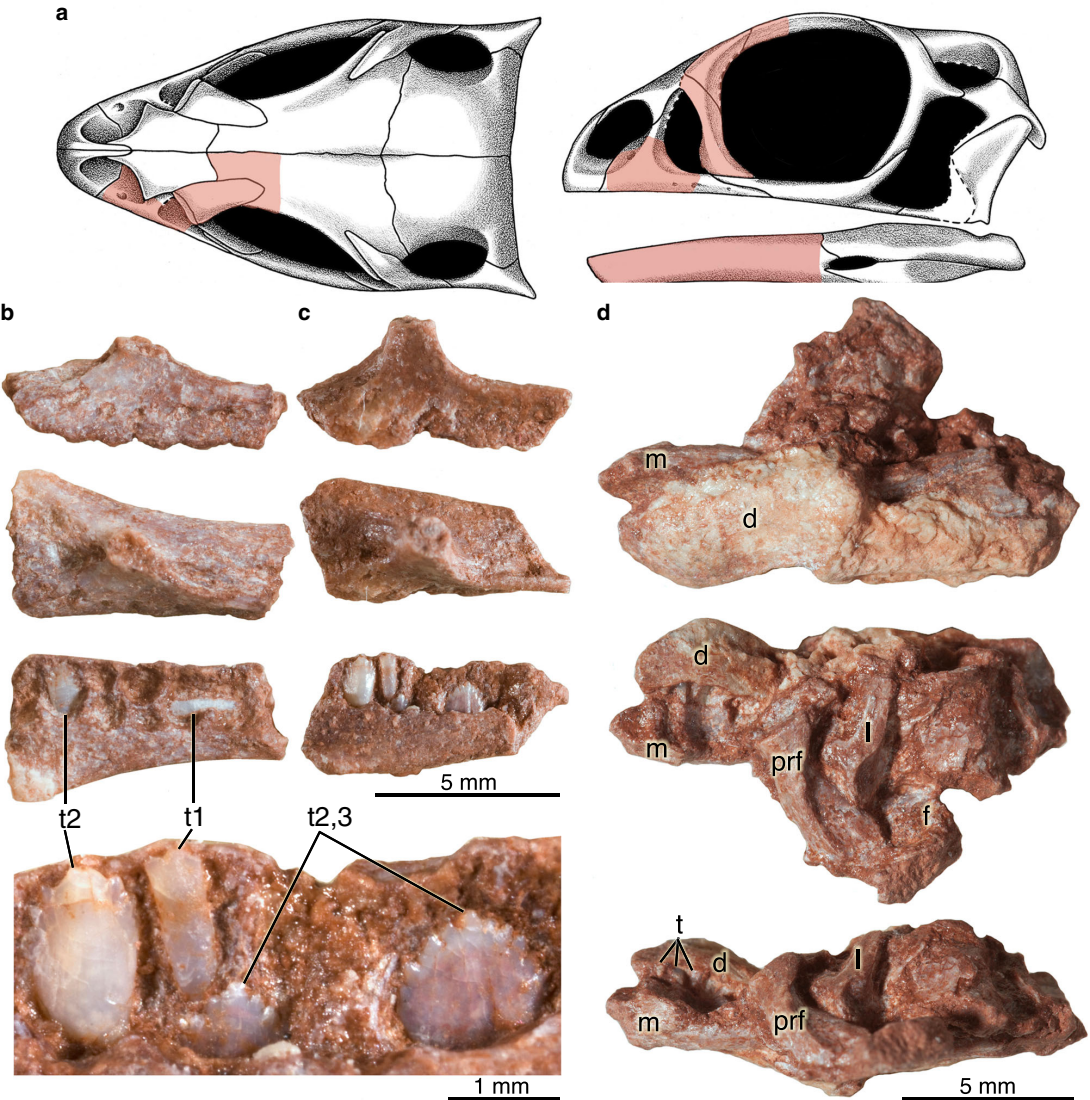
Embryonic cranial materials of sauropodomorph dinosaur *Lufengosaurus* (Chuxiong Prefectural Museum C2019 2A233). **a** Reconstructed embryonic skull^18,19^ in dorsal and lateral views, showing preserved portions of the skull. **b** Left maxilla with two preserved teeth in lateral, dorsal, and ventral views. **c** Left maxilla with four preserved teeth in lateral, dorsal, and ventromedial views, with enlarged view of four maxillary teeth, showing crown anatomy in two distinct generations of unerupted teeth. **d** Cranial fragment showing maxilla and dentary bones in partial lateral, medial, and dorsal views. Abbreviations: d, dentary; f, frontal; l, lacrimal; m, maxilla; prf, prefrontal; t, teeth; t1, older embryonic teeth; t2-3, younger replacement teeth. Note that t2-3 replacement teeth may represent differing generations, with the tooth to the immediate left of the t1 tooth is significantly older than the two other teeth that are just forming in this individual.

**Fig. 2:**
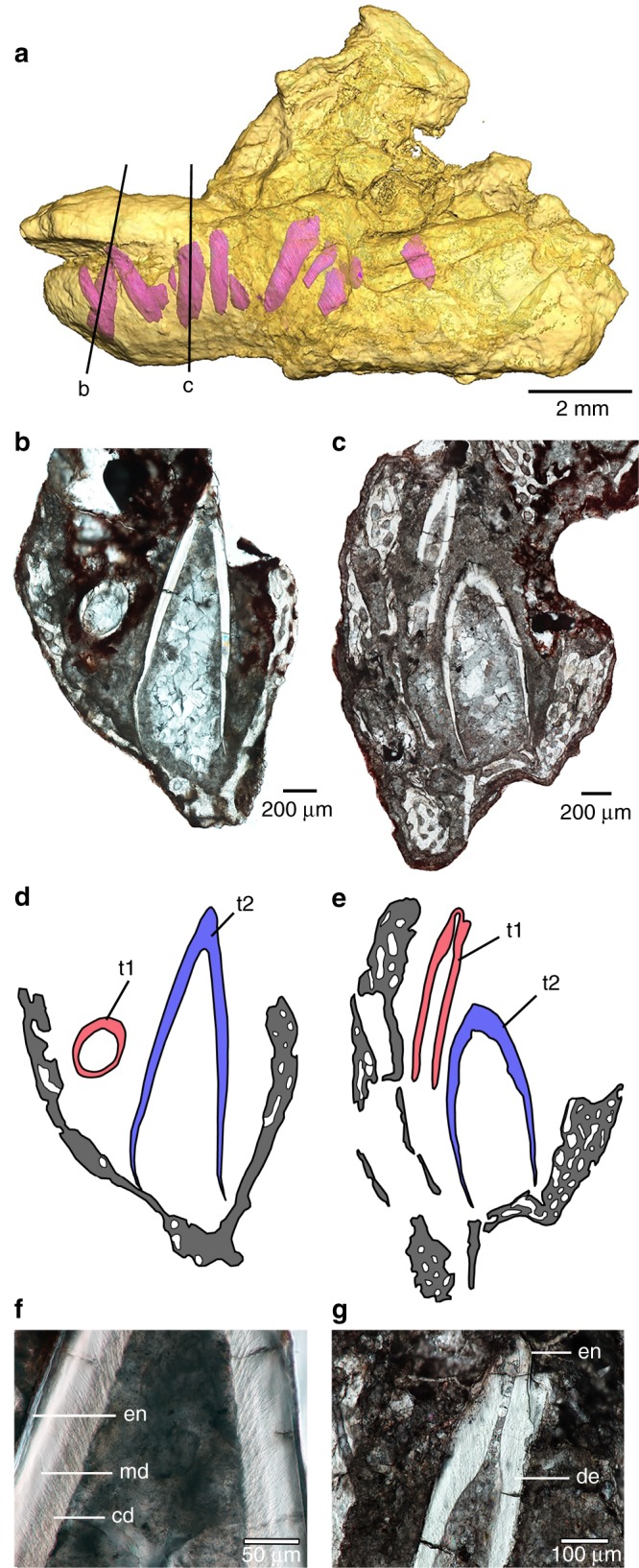
Tooth development in the stem sauropod embryos of *Lufengosaurus* (C2019 2A233). **a** Three-dimensional model of skull fragment (C2019 2A233) from computed tomographic scans showing embryonic teeth (pink) and surrounding jawbones (yellow). **b** Thin section of the anterior tooth showing a small, older tooth (t1) and a much larger replacement tooth (t2) forming lingually (lingual is to the right). Both teeth develop within a trough in the dentary. **c** Thin section at a more distal tooth position showing two generations of teeth (lingual is to the right). **d** Drawing of thin section in (**b**) showing size and position of smaller, older tooth (t1 in red) and much larger replacement tooth (t2 in blue). Gray indicates jawbone. **e** Drawing of thin section in (**c**) showing implantation of older tooth (red) and the relative size of the larger replacement tooth (blue). **f** Enlarged image of replacement tooth (t2) in (**b**) showing dental tissues. Note the preservation of dentinal tubules in the circumpulpal dentine. **g** Enlarged image of older tooth (t1) in (**c**) showing dental tissues. Abbreviations: cd, circumpulpal dentine; de, dentine; en, enamel; md, mantle dentine; t1, older tooth; t2, replacement tooth.

The embryonic maxillae and dentaries represent several individuals of the sauropodomorph *Lufengosaurus*^14,17^. The two isolated maxillary fragments (Fig. 1b, c) included in this study are similar in size, preserve approximately the anterior half of the tooth-bearing region of the bone, and have spaces for five teeth. A third embryonic skull specimen includes not only the maxilla and dentary but also other parts of the snout and may represent a slightly more advanced embryonic stage than the two isolated maxillae based on size and on tooth developmental stages^16^ (Fig. 2 and Supplementary Movie 1). The left maxillary fragment (Fig. 1d) in this third specimen does not preserve any teeth, but the left dentary is nearly complete, preserving at least nine tooth positions and numerous teeth. We employed a combination of HRµCT scanning (Fig. 2a) for positional information, and thin sectioning (Fig. 2b–g and Supplementary Fig. 1) for detailed tissue information about the dentary bone and its teeth.

### Embryonic tooth development in *Lufengosaurus*

We observed at least two, likely three morphologically distinct generations of teeth in single specimens of *Lufengosaurus* embryos. The younger generations, t2 and t3, resemble the teeth of the adults in being labiolingually flattened crowns with large denticles (Fig. 2). By comparison, the older tooth generation teeth (t1) are slender pegs with no crown expansion, more closely resembling resorptive or transitional teeth in modern crocodilians^20–22^. Each tooth-bearing maxilla preserves an older tooth generation, t1, and up to four such teeth are also found in the dentary. These older teeth are all associated with a broad-crowned t2 replacement tooth lingually, suggesting that the t1 generation of teeth were rapidly replaced with a more adult-like tooth morphology. These t1 teeth are similar to the functional teeth of certain titanosaur and diplodocoid sauropods^1,23,24^. These teeth also lack the lingual median ridge that is present in the functional teeth of basal sauropodomorphs, the presence of which is the ancestral condition for dinosaurs^25^.

In contrast to the embryonic dentitions found in other dinosaurs that preserve teeth^6,10,26^, none of these teeth appear to have erupted, even though they are preserved in the fossils at various stages of development (Fig. 2c, e). Although apparently fully formed, with well-preserved enamel, mantle dentine, and orthodentine with dentinal tubules (Fig. 2f), the younger teeth do not extend beyond the labial edge of the maxilla and extend only slightly beyond the edge of the dentary, indicating that they were likely still covered by the gingiva in the developing embryo.

Additionally, HRµCT data indicate that none of the embryonic teeth of *Lufengosaurus* were fused to the jawbone, a tooth attachment feature they share with other dinosaurs^4,10^. One of the pencil-shaped t1 teeth is preserved lying horizontally in the empty crypt of one maxilla (Fig. 1b), probably after the soft tissues suspending it in place decomposed. The same condition is apparent in the dentary, where many of the teeth have moved slightly out of position post mortem, but do not show any evidence of root resorption (Fig. 2b–e). These observations, as well as the presence of two generations of teeth at many tooth positions seen in both the HRµCT data and histological thin sections, suggest that the youngest generation of teeth was well-developed, supported by soft tissue, and likely replaced at least once before hatching. The mechanism of tooth replacement is still unclear; despite the abundance of t1 and t2 teeth, thin sections did not reveal any evidence of root resorption in the embryos.

### Comparisons with *Alligator*

**Fig. 3:**
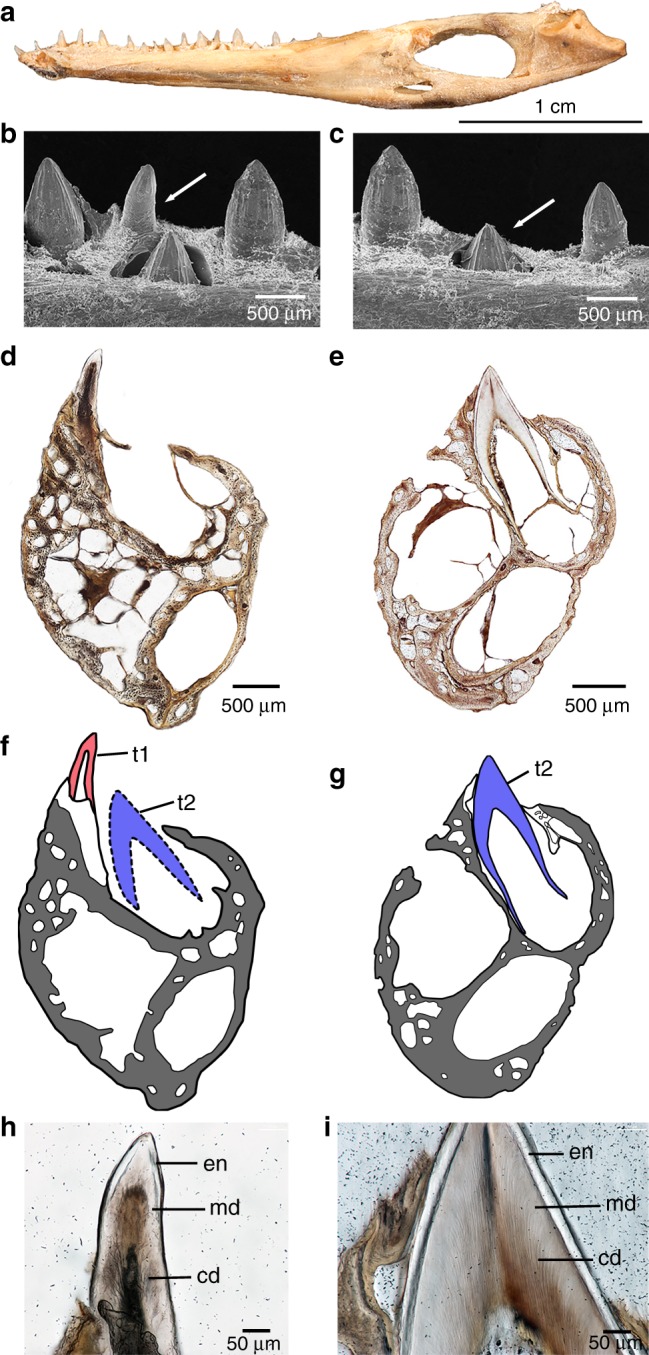
Tooth development in a 1 day-old *Alligator mississippiensis* hatchling (ROM R7964). **a** Right dentary of ROM R7964. **b** Scanning electronic microscopic image of the small embryonic tooth sectioned in (**d**) (white arrow) showing larger replacement tooth visible in lingual view. **c** Scanning electronic microscopic image of replacement tooth sectioned in (**e**) (white arrow) in lingual view. **d** Thin section of the left dentary of ROM R7964 showing implantation of small, embryonic tooth. The large space lingual to the tooth housed the replacement tooth in (**b**) that fell out during thin sectioning. **e** Thin section taken two tooth positions distally from (**d**) showing size of erupting replacement tooth. **f** Drawing of (**d**) showing mode of implantation of embryonic tooth (t1 in red) and position of replacement tooth (t2 in blue). Gray and white indicate jawbone and attachment tissues respectively. **g** Drawing of erupting tooth in (**e**). **h** Enlarged image of embryonic tooth in (**d**) showing dental tissues. **i** Enlarged image of replacement tooth in (**e**) showing dental tissues. Abbreviations: cd, circumpulpal dentine showing dentinal tubules; de, dentine; en, enamel; md, mantle dentine; t1, older embryonic tooth; t2, younger replacement tooth.

Comparing the *Lufengosaurus* embryos with the teeth of a 1-day-old *Alligator* hatchling (Fig. 3) revealed similar patterns of tooth development, where successive generations of teeth occupy a single large crypt with no intervening jaw or socket bone tissues. In extant *Alligator* embryos and hatchlings, the oldest teeth have similar shapes to, but are much smaller than, the next generation of teeth and do not erupt into the oral cavity^20^. In early *Alligator* hatchlings, the erupted teeth are only attached to the labial wall of the jawbone, effectively forming a pleurodont implantation (Fig. 3d)^27^. This occurs in *Alligator* embryos because the replacement and functional teeth occupy the same crypt, with the larger, younger replacement tooth developing lingual to the older tooth (Fig. 3d). The lingual wall of the jaw therefore does not contribute to tooth attachment in the earliest functional teeth in *Alligator* hatchlings (Fig. 3b, d). However, after hatching, successive generations of replacement teeth gradually migrate to a sub-dental position, beneath the functional tooth, in typical thecodont fashion^27^. This pattern is retained in adult *Alligator*, and periodontal tissues completely surround the functional teeth^22,28^.

**Fig. 4:**
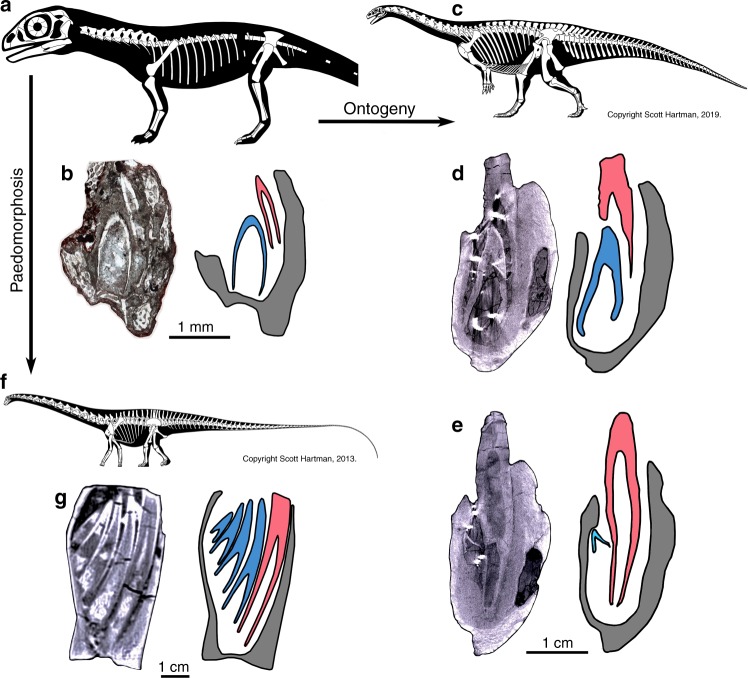
Ontogeny and paedomorphosis in sauropodomorph dinosaurs. **a** Reconstructed embryonic skeleton of *Lufengosaurus*. **b** Thin section through two equally tall embryonic teeth in the wide alveolar trough showing two generations of teeth without evidence of replacement. **c** Reconstruction of adult *Lufengosaurus*, CVP 148-6. **d**, **e** CT scan of adult *Lufengosaurus* dentary showing narrow alveolar trough and two stages of tooth replacement (old tooth in red and new tooth in blue) one at the initial stage of development, and the second showing tooth root resorption caused by the growth of new tooth. **f** Reconstruction of adult *Diplodocus*. **g** Computed tomographic scan of adult *Diplodocus* showing multiple generations of teeth forming in wide alveolar trough without root resorption. *Diplodocus* YMP 4677 (modified from Figure 2, D’Emic et al.^1^).

This ontogenetic shift is clearly seen in *Lufengosaurus* as well. The first generation of embryonic teeth are undersized compared to the alveolar trough, and lie in a single large crypt in which both younger and older tooth generations are aligned labiolingually, with no intervening mineralized tissues^29^. The oldest teeth (t1) line the labial wall of the jaw, whereas the much larger (t2) teeth develop lingually without causing root resorption to t1 teeth (Fig. 4).

### Comparisons with neosauropods

Tooth replacement in later ontogenetic stages of *Lufengosaurus* more closely resemble typical tooth replacement in non-neosauropodomorph dinosaurs^2,4^. In a hatchling specimen of *Lufengosaurus*, the replacement teeth are found in a sub-dental position, and root resorption is present (Supplementary Fig. 1). Similarly, in the adult *Lufengosaurus*^15^ and other basal sauropodomorphs^30,31^, alveolar bone surrounds the older tooth, the replacement teeth develop lingually, gradually migrate beneath the functional tooth, and eventually cause apical root resorption and tooth shedding (Fig. 4d, e)^14^.

However, embryonic tooth development and replacement in *Lufengosaurus* embryos more closely resembles the dentition in several adult neosauropods than adult, basal sauropodomorphs. For example, in *Diplodocus*, the undersized functional teeth are restricted to the labial margins of large troughs in the jawbones, replacement teeth form along the lingual margin, and the replacement teeth migrate labially without causing root resorption to older tooth generations (Fig. 4g). Similar patterns of tooth development have been observed in several other neosauropods, including *Nigersaurus, Camarasaurus*, and some titanosaurs^1^. We observed similar phenomena in the *Lufengosaurus* embryos, where the t1 teeth are undersized relative to the alveolar trough and there is no initial resorption of t1 teeth (Fig. 4b).

### Geometric morphometric analyses

In order to investigate the degree of shape variation in teeth of *Lufengosaurus* embryos, we conducted three separate geometric morphometric analyses of these teeth (one for tooth outline, one for crown cross-section, and one for root cross-section), comparing them to other basal and crown sauropodomorphs for similarities.

**Fig. 5:**
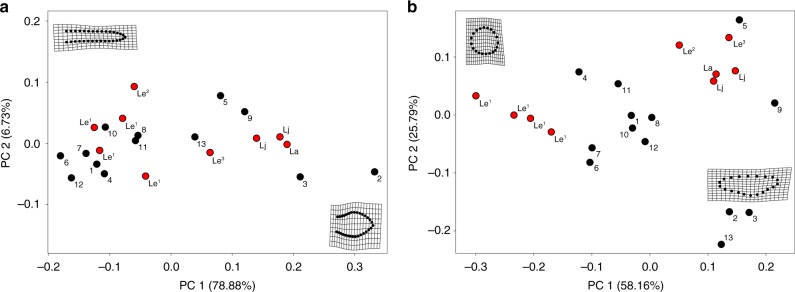
Geometric morphometric analysis of embryonic and adult sauropodomorph dentition. **a** PC1 versus PC2 of tooth outline (85.61% of the total variation). **b** PC1 versus PC2 of crown cross-section (83.95% of the total variation). Deformation grids represent morphologies at the positive and negative extremes of PC1 in each plot. Numbers indicate: 1, *Abydosaurus*; 2, *Amygdalodon*; 3, *Bellusaurus*; 4, *Bonitasaura*; 5, *Camarasaurus*; 6 and 7, *Diplodocus*; 8, Kem Kem Titanosaur; 9, *Mamenchisaurus*; 10, MML PV 1030; 11, *Nemegtosaurus*; 12, *Rapetosaurus*; 13, *Shunosaurus*. Letters indicate: Le^1^, *Lufengosaurus* embryonic tooth stage 1; Le^2^, *Lufengosaurus* embryonic tooth stage 2; Le^3^, *Lufengosaurus* embryonic tooth stage 3, data taken from the mandible; Lj, *Lufengosaurus* juvenile, data taken from CVP 148-4; La, *Lufengosaurus* adult, data taken from CVP 148-6.

PC axes 1 and 2 from the analysis of the tooth crown outlines (Fig. 5a) cumulatively represent 85.61% of the variation in tooth shape, and the remaining axes account for little variation (less than or equal to 5% each). PC1 positive values represent teeth that are relatively short and broad, whereas PC1 negative values represent teeth that are elongated and thin. The pencil-like teeth of titanosaurs and diplodocoids, along with the embryonic *Lufengosaurus* teeth from the mandible (t1-3, Le^1–3^ in Fig. 5 and Supplementary Figs. 2 and 3), plot towards PC1 negative, whereas the leaf-shaped teeth of the juvenile (Lj) and adult *Lufengosaurus* (La) plot towards PC1 positive, along with the teeth of *Amygdalodon*, *Bellusaurus*, *Camarasaurus*, *Mamenchisaurus*, and *Shunosaurus*. PC2 represents symmetry along the long axis of the teeth, where PC2 positive values represent more asymmetric teeth and PC2 negative values represent more symmetric teeth. There are no apparent differences between the pencil-like teeth and leaf-shaped teeth along PC2, although the *Lufengosaurus* embryonic specimens show the greatest variation along this axis.

PC axes 1–3 of the principal component analyses (PCA) of tooth crown cross-section cumulatively represent 93.05% of the variation in crown cross-section, and the remaining axes account for little variation (less than 5% each). PC1 encompasses the majority of the variation in crown cross-sectional shape, and represents circularity of the crown cross-section (Fig. 5b). PC1 positive values represent crowns that are labiolingually compressed, and PC1 negative values represent crowns that are nearly circular in cross-section. The embryonic (t2 and t3), juvenile, and adult *Lufengosaurus* crowns, along with *Amygdalodon*, *Bellusaurus*, *Camarasaurus*, *Mamenchisaurus*, and *Shunosaurus*, plot towards PC1 positive, whereas the circular embryonic *Lufengosaurus* crowns (t1) plot towards PC1 negative. The titanosaur and diplodocoid crowns plot more positively along PC1 than the t1 embryonic *Lufengosaurus* teeth due to their slightly elliptical cross-section. PC2 represents concavity of lingual surface and the presence of the lingual median ridge. PC2 positive values represent teeth with little to no lingual concavity and a prominent lingual median ridge, and PC2 negative values represent teeth with a prominent lingual concavity and no lingual median ridge. The t2 and t3 teeth, along with *Camarasaurus*, plot towards PC2 positive, while the teeth of *Amygdalodon*, *Bellusaurus*, and *Shunosaurus* plot towards PC2 negative. The remainder of the teeth are either elliptical or circular in cross-section, therefore lacking both the lingual concavity and lingual median ridge, and plot towards zero along PC2.

PC3 (9.10% of the total variation) represents asymmetry of the crown cross-sections along the long axis of the tooth, where PC3 positive values represent crowns that are more asymmetrical, and PC3 negative values represent crowns that are more symmetrical (Supplementary Fig. 2). The broad-shaped teeth possess greater variation along PC3 than the pencil-like teeth, but there are no apparent differences between these groups along PC3.

PC axes 1 and 2 of the PCA of root cross-section cumulatively represent 90.21% of the variation in root cross-section shape (Supplementary Fig. 3), and the remaining axes account for little variation (less than 5% each). The PCA of root cross-section provides some insight into shape variation in the *Lufengosaurus* tooth roots and compare them to titanosaurs and diplodocoids. PC1 encompasses the majority of the variation in root cross-sectional shape (75.90%), and represents circularity of the root cross-section, similar to the PCA of crown cross-section. PC1 positive values represents roots that are labiolingually compressed and are therefore elliptical in cross-section, and PC1 negative values represent roots that are nearly circular in cross-section. The juvenile and adult *Lufengosaurus* roots, along with *Camarasaurus*, plot towards PC1 positive, while the circular t1, t2, and t3 (Le^1–3^ in Supplementary Fig. 3) *Lufengosaurus* crowns plot towards PC1 negative, along with the remainder of the neosauropods. PC2 (14.31% of the total variation) represents asymmetry of the root cross-sections along the long axis of the tooth, where PC2 positive values represent roots that are more symmetrical, and PC2 negative values represent roots that are more asymmetrical. The pencil-like neosauropod teeth possess greater variation along PC2 than the *Lufengosaurus* teeth, but there are no apparent differences between these groups along PC2.

The results from these morphometric analyses (Fig. 5 and Supplementary Figs. 2 and 3) highlight the shape variation in successive generations of embryonic *Lufengosaurus* teeth. The t1 teeth are similar to the pencil-like teeth of titanosaurs and diplodocoids, while the t2 and t3 teeth are intermediates between the t1 and the juvenile/adult condition. These t2 and t3 teeth are labiolingually compressed with a lingual median ridge, similar to the juvenile and adult condition in *Lufengosaurus*, but possess round roots, similar to the t1 and titanosaur/diplodocoid teeth.

## Discussion

Two embryonic features of the dentition of the basal sauropodomorph *Lufengosaurus* shed light on the development and potentially the origin of the dentition of certain neosauropods, including diplodocoids and titanosaurs. In *Lufengosaurus* embryos, the oldest teeth (t1) are small relative to the alveolar trough in which they are implanted and are only in contact with the labial wall of the jaw (Fig. 2). As a result, the much larger replacement teeth form lingual to their predecessors and do not appear to cause resorption of the roots of the older teeth (Fig. 4b). Embryonic *Lufengosaurus* teeth are therefore implanted in a pleurodont fashion, similar to hatchling alligators (Fig. 3)^27^, and even some sauropods^32^. In neosauropods, as many as 4–8 generations of teeth are present at each tooth position within a single large trough, and there are no mineralized periodontal tissues between successive generations of teeth, except for a thin layer of cementum coating the tooth roots^1,12,23^.

Recent histological studies have suggested that sauropods were truly thecodont based on the presence of the stereotypically mammalian complement of dental attachment tissues^12^, but the size discrepancy between the teeth and the jaws means that functional diplodocid teeth are implanted in a similar, pleurodont fashion to the t1 teeth of the embryonic specimens of *Lufengosaurus*. The alveolar trough in diplodocoids is extremely large relative to the widths of the individual teeth and therefore creates ample space to hold multiple generations of teeth at a single tooth position without causing root resorption (Fig. 4g). Conversely, adult stem sauropod tooth implantation and replacement are more typical of other early dinosaurs^29^. The teeth approach a thecodont condition in which functional teeth are more symmetrically implanted within the alveolus (Fig. 4d, e). As a result, replacement teeth in adult stem sauropods form slightly lingual to their predecessors and cause root resorption even at comparatively early stages of replacement tooth development.

The second feature of great interest relates to ontogenetic variation in the shapes of the teeth in *Lufengosaurus* embryos. The adult dentition of diplodocoids and many derived titanosaurs convergently evolved into smaller and more pencil-shaped teeth^1,33–35^. Both the development and the pencil-shaped morphology of the adult dentition reflect features that characterize the embryonic stages of tooth development in early sauropodomorphs, as shown here.

Of all of the embryonic teeth, the t1 teeth exhibit the strongest similarity to the titanosaurs and diplodocoids in crown outline. The t2 teeth also exhibit strong similarity in outline to the titanosaurs and diplodocoids, although the t2 teeth are more asymmetrical (Fig. 5). The t3 teeth are intermediate in shape between the former developmental stages and the teeth of the juvenile and adult, where they are broader than the pencil-like t1 and t2 teeth, but not as broad as the functional teeth of juvenile and adult *Lufengosaurus*.

It is important to note that the t1 crowns of embryonic *Lufengosaurus* are more similar in cross-sectional shape to the teeth of titanosaurs and diplodocoids than to the teeth of its later developmental stages. The strong difference in cross-sectional shape between t1 crowns and later generations of teeth is due primarily to the presence of a lingual median ridge in these later developmental stages that is absent in the t1 generation. Patterns in root cross-sectional shape differ from crown cross-section, where the *Lufengosaurus* t1, t2, and t3 roots plot among the pencil-like teeth of the neosauropods, whereas the juvenile and adult *Lufengosaurus* teeth plot towards PC1 positive, along with *Camarasaurus*. Taken together, the PCAs of tooth outline and crown and root cross-sections suggest that the t1 teeth are similar to the pencil-like teeth of titanosaurs and diplodocoids, and the t2 and t3 teeth are intermediate between the t1 and juvenile/adult condition. The t2 and t3 teeth are labiolingually compressed with a lingual median ridge, similar to the juvenile and adult condition, but possess round roots, similar to the t1 and titanosaur/diplodocoid teeth.

These similarities in tooth development between some derived neosauropods and the embryonic stages of tooth development in early stem sauropods lead us to hypothesize that heterochrony may explain how these two elements of the complex dentitions of neosauropods evolved, with some diplodocoids and titanosaurs independently developing dental complexes composed of several relatively small teeth within a large dental crypt. It has been previously proposed that paedomorphosis^36^ may have played a role in the evolution of sauropod quadrupedality^19,37^, and dissociated heterochrony has also been hypothesized for certain cranial and vertebral morphologies in diplodocid sauropods^38^. It has also been proposed that the retention of the embryonic and early juvenile body proportions in the adult sauropods (through paedomorphosis) may have been a contributing factor in the evolution of large size in this clade of herbivores^17,19^. Here we have provided new evidence that paedomorphosis, through the retention of embryonic developmental patterns, may have been pervasive in sauropodomorph evolution, being potentially also associated with the evolution of the derived cropping mechanism of certain sauropods, critical to their feeding behaviors. We highlight two potentially independent features—retention of simple embryonic tooth morphology and an asymmetrical form of tooth development and replacement—as products of the truncation of dental development before attaining the ancestral adult condition. Additionally, retention of embryonic tooth implantation, and co-occurrence of multiple generations of teeth positioned in a labio-lingual configuration may have permitted the more rapid accumulation of successive generations of teeth that comprise the dental battery of some neosauropods.

Paedomorphosis provides a mechanistic explanation for the convergent evolution of small, pencil-like teeth in diplodocoid and titanosaurid sauropods^24,39^. As is the case in other tetrapod groups, convergent developmental perturbations may result in similar morphologies^30^. Although often associated with small body size^40^, paedomorphosis and the retention of embryonic features in adults is here shown to be also associated with evolutionary changes in organisms of very large body mass^41^. Thus, the evidence presented here demonstrates the essential role of paedomorphosis in events leading to the success of the largest land-dwelling vertebrates of all time.

## Methods

### Preparation and paleohistology

Fossil preparation was done manually under a dissecting microscope. Thin sections were prepared on plastic slides using an isomet 1000 wafer blade saw. The sections were then ground down to thicknesses ranging from 30 to 50 µm using a Hillquist grinding machine, as well as 600- to 1000-grit silicon carbine powder. Thin sections were photographed using a Nikon AZ100 microscope.

### High-resolution micro-computed tomography and visualization

The scans of the embryonic materials were performed on a SkyScan 1173 system (Bruker, Belgium), using 110 kV, 72 µA, 500 ms exposure, a rotation step of 0.2, and an isometric voxel resolution of 6.04 µm. Scan data were imported in Amira v.5 (FEI, Hillsboro, OR) as a sequence of stacked tiffs. Individual teeth were digitally segmented by applying the LabelFields module to the data, and then were visualized by applying the SurfaceGen and SurfaceView modules to the segmented data.

### Morphometric analyses

Three separate two-dimensional (2D) morphometric analyses were performed in this study (one for tooth outline, one for crown cross-section, and one for root cross-section) to describe the variation in tooth outline, and crown and root cross-sections separately. The analyses were separated because tooth outline and tooth cross-section cannot be viewed simultaneously, and a single 2D morphometric analysis therefore would be unable to capture these shapes at the same time. Three-dimensional (3D) models were not available for all taxa, and therefore 3D morphometrics could not be implemented to capture tooth outline and cross-sectional shape for all taxa in the dataset.

Embryonic *Lufengosaurus* teeth were imaged in labial view for analysis, along with teeth from juvenile and adult *Lufengosaurus*, and several additional eusauropod taxa for comparison. All teeth were digitally traced to produce an outline of the tooth, and cross-sections mid-way down the apical–basal length of the crown and mid-way down the length of the root were imaged and traced to produce cross-sectional shapes. Crown outlines and crown cross-sections were also taken from the literature (e.g., refs. ^18,24,30,32,42–45^). Since root cross-sections were not available for all taxa in this analysis, the bulk of the interpretations are based on crown outline and crown cross-section.

Tooth outline and cross-section outlines were loaded separately in ImageJ 1.52a for landmarking. Thirty-one landmarks were placed along the outline of the tooth crowns. The first and 31st landmarks were designated as fixed landmarks, and were placed on the basal-most extent of the enamel. The 16th landmark was also designated as a fixed landmark, placed at the apex of the tooth (defined as the most acute angle of the tooth crown; D’Amore^46^). Twenty-eight evenly spaced semilandmarks were placed along the outline of the teeth, 14 between landmarks 1 and 16, and 14 between landmarks 16 and 31. Twenty landmarks were placed along the tooth cross-sections, with 1 fixed landmark placed on the labial-most edge, and 19 evenly spaced semilandmarks placed counter-clockwise around the tooth.

Tooth outline and tooth cross-section landmarks were separately imported into RStudio 1.2.5019 as plain text files (*.txt) for processing. Landmarks were aligned and scaled using the General Procrustes Alignment function “gpagen” in the R package *geomorph* 3.1.3 (Adams et al.^47^). Semilandmarks were allowed to slide between their neighboring landmarks to minimize bending energy. Aligned outline and cross-section landmarks were then projected into separate morphospaces using principal component analyses (PCA) from the *geomorph* function “plotTangentSpace” to visualize outline and cross-sectional shape variation across the dataset.

### Reporting summary

Further information on research design is available in the Nature Research Reporting Summary linked to this article.

## Supplementary information

Supplementary Information

Reporting Summary

Description of Additional Supplementary Files

Supplementary Movie 1

## Data Availability

The fossil materials C2019 2A233 are deposited in the collections of the Chuxiong Prefectural Museum, Chuxiong, Yunnan Province, China, CVP 148-6 is deposited at the Field Museum, Chicago, and YMP 4677 is deposited at the Yale Peabody Museum, New Haven, CT, USA. The extant crocodilian material used in this study (ROM R7964) is part of the Vertebrate Paleontological Collections of the Royal Ontario Museum, Toronto, ON, Canada. All raw CT data are available upon request at the respective museums. The morphometric data are available from the corresponding author upon reasonable request.
