# Supplementary material for: Early Jurassic dinosaur fetal dental development and its significance for the evolution of sauropod dentition
Source: Nat Commun. 2020 May 7;11:2240. doi: 10.1038/s41467-020-16045-7 (PMC7206009; doi:10.1038/s41467-020-16045-7)
Supplement: Supplementary file 1 — Supplementary Information [file 41467_2020_16045_MOESM1_ESM.pdf]

Supplementary Information

Early Jurassic dinosaur fetal development and its significance for the evolution of sauropod dentition

Robert R. Reisz[1,2,3*], Aaron R.H. LeBlanc[4], Hillary Maddin[5], Thomas W. Dudgeon[5], Diane Scott[2], Timothy Huang[1,3], Jun Chen[1], Chuan-Mu Chen[3], Shiming Zhong[6]

[1] Dinosaur Evolution Research Centre and International Centre of Future Science, Jilin University, Changchun, Jilin Province, China

[2] Department of Biology, University of Toronto Mississauga, Mississauga, Ontario L5L 1C6, Canada.

[3] National Chung Hsing University, Taichung 40227, Taiwan.

[4] Department of Biological Sciences, University of Alberta, Edmonton, Alberta, Canada T6G 2E9

[5] Department of Earth Sciences, Carleton University, Ottawa, K1S 5B6, Canada.

[6] Chuxiong Prefectural Museum 675000 Chuxiong, Yunnan, China.

Supplementary Figures

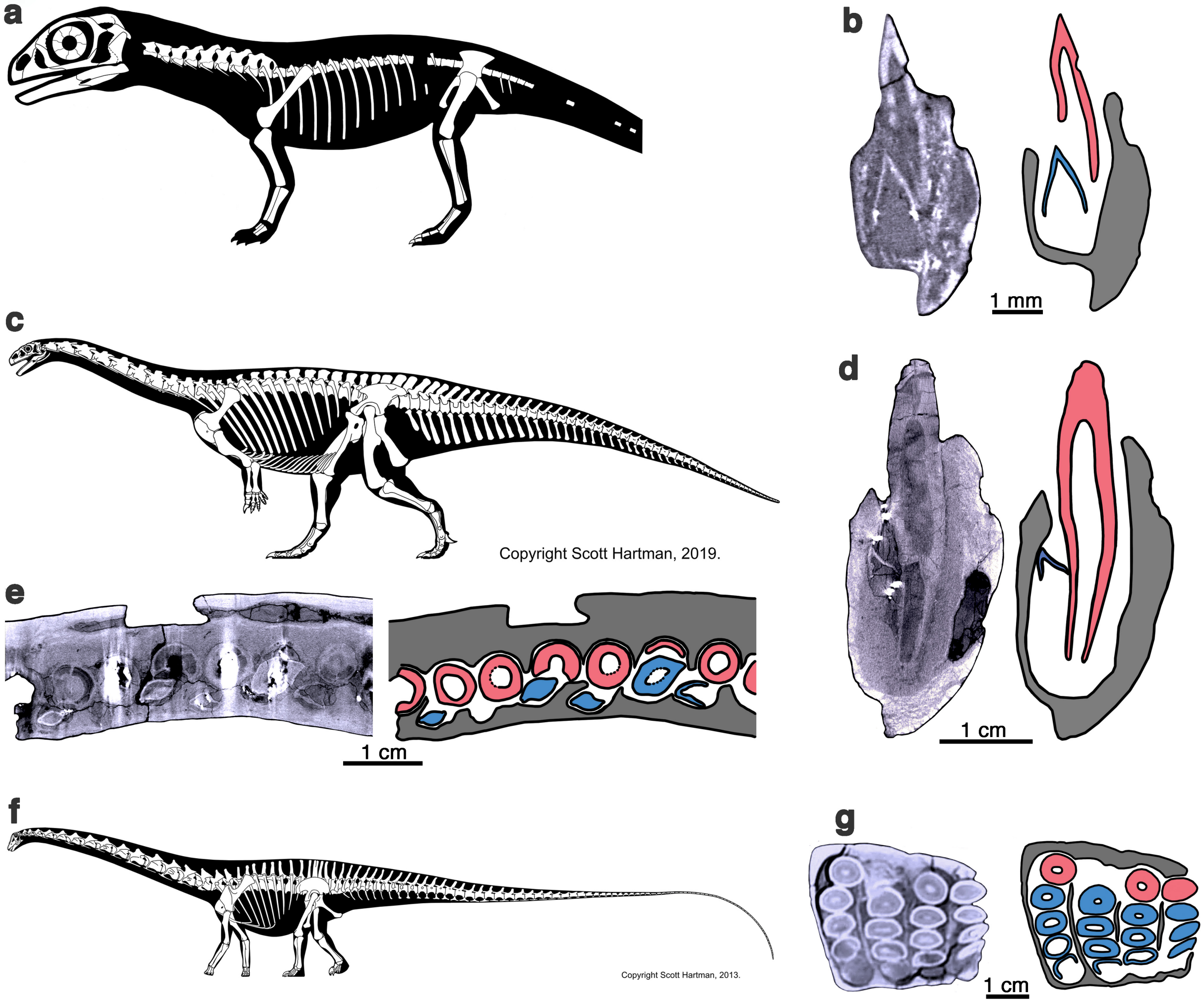

Supplementary Fig. 1 Additional data on tooth development and tooth replacement is sauropodomorph dinosaurs. a, b, Skeletal reconstruction of presumed hatchling of *Lufengosaurus,* with cranial and dental data provided by the hatchling CUP 2037 (Catholic University of Peking, now housed in the Field Museum of Natural History), previously *Fulengia youngi*[18]. Note that by this stage of its ontogeny, the older tooth is fully erupted and functional (in red), and the replacement tooth is ventral to it (in blue), and has penetrated the pulp cavity through root resorption if the functional tooth. **c-e,** Skeletal reconstruction of adult *Lufengosaurus* for references, and CT data of dentary bone showing tooth development and replacement patterns that are typical of basal adult dinosaurs. **d,** Note the formation of a new tooth below the edge of the dentary bone, lingually. At this early stage of tooth development root resorption has not started yet, but in **e,** various stages of tooth development and root resorption are observed in this transverse CT sectional view of the dentary, with older teeth in red and replacement teeth in blue. **f, g,** Skeletal reconstruction of adult *Diplodocus* for reference, and dental battery in transverse CT section, showing functional teeth in red, replacement teeth in blue at various stages of development. Note that there is no evidence of resorption between teeth.

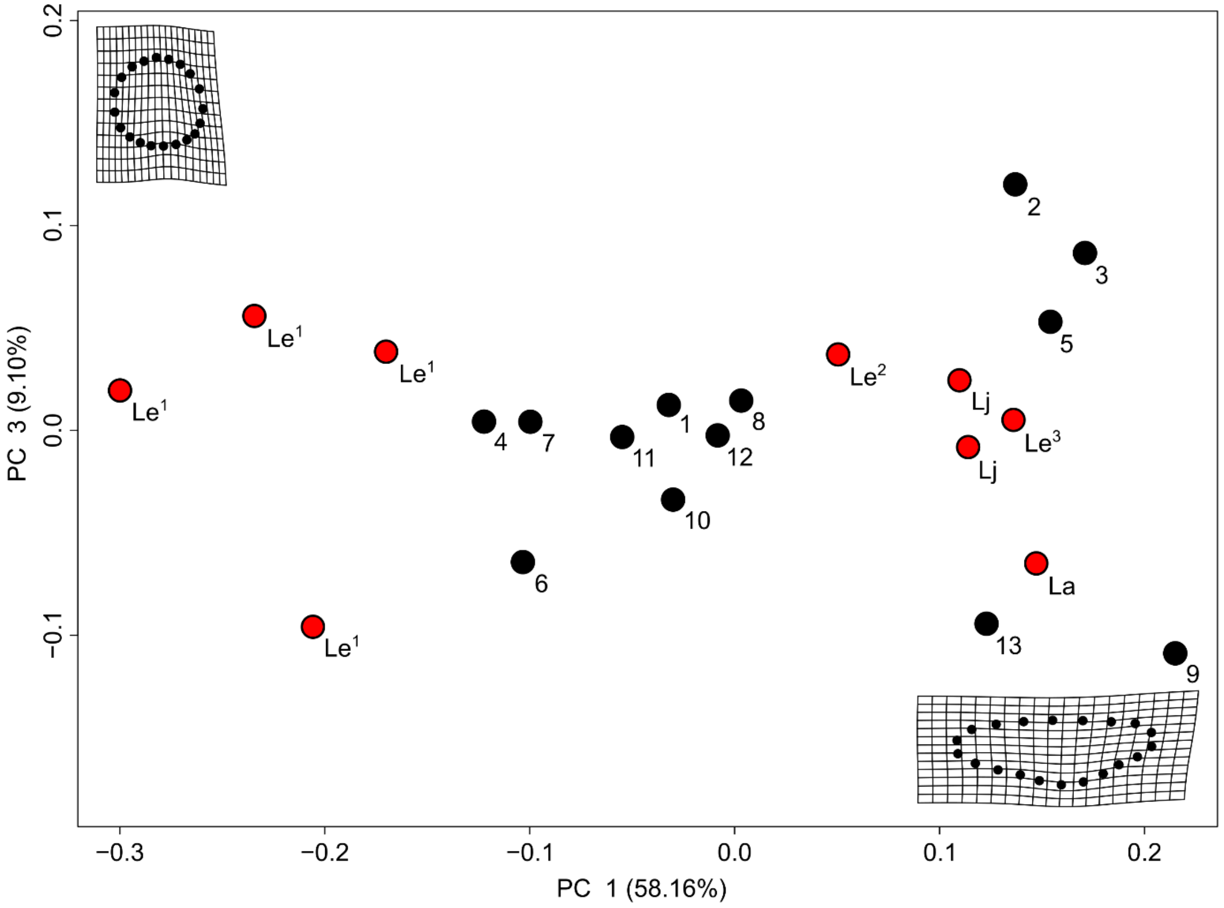

Supplementary Fig. 2 Geometric morphometric analysis of embryonic and adult sauropodomorph dentition. PC1 versus PC3 of crown cross-section (90.21% of the total variation). Deformation grids represent morphologies at the positive and negative extremes of PC1 in each plot. Numbers indicate: 1, *Abydosaurus*; 2, *Amygdalodon*; 3, *Bellusaurus*; 4, *Bonitasaura*; 5, *Camarasaurus*; 6 and 7, *Diplodocus*; 8, Kem Kem Titanosaur; 9, *Mamenchisaurus*; 10, MML PV 1030; 11, *Nemegtosaurus*; 12, *Rapetosaurus*; 13, *Shunosaurus*. Letters indicate: Le^{1}, *Lufengosaurus* embryonic t1; Le^{2}, *Lufengosaurus* embryonic t2; Le^{3}, *Lufengosaurus* embryonic t3; Lj, *Lufengosaurus* juvenile; La, *Lufengosaurus* adult.

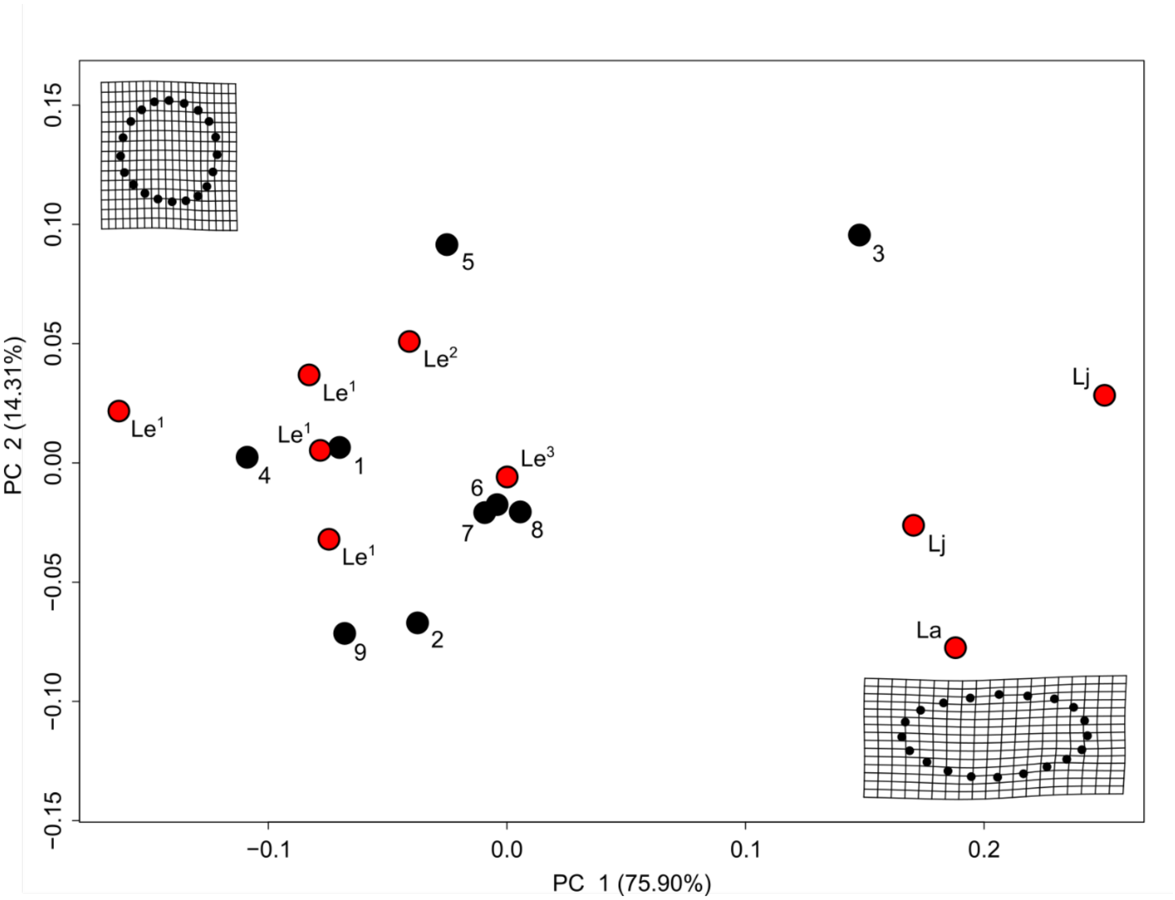

Supplementary Fig. 3 Geometric morphometric analysis of embryonic and adult sauropodomorph dentition. PC1 versus PC2 of root cross-section (90.21% of the total variation). Deformation grids represent morphologies at the positive and negative extremes of PC1 in each plot. Numbers indicate: 1, *Abydosaurus*; 2, *Bonitasaura*; 3, *Camarasaurus*; 4 and 5, *Diplodocus*; 6, Kem Kem Titanosaur; 7, MML PV 1030; 8, *Nemegtosaurus*; 9, *Rapetosaurus*. Letters indicate: Le^1, *Lufengosaurus* embryonic t1; Le^2, *Lufengosaurus* embryonic t2; Le^3, *Lufengosaurus* embryonic t3; Lj, *Lufengosaurus* juvenile; La, *Lufengosaurus* adult.
