# Supplementary material for: Early Jurassic dinosaur fetal dental development and its significance for the evolution of sauropod dentition
Source: Nat Commun. 2020 May 7;11:2240. doi: 10.1038/s41467-020-16045-7 (PMC7206009; doi:10.1038/s41467-020-16045-7)
Supplement: Supplementary file 2 — Reporting Summary [file 41467_2020_16045_MOESM2_ESM.pdf]

nature research

Corresponding author(s): Robert Reisz

Last updated by author(s): April 6, 2020

Reporting Summary

Nature Research wishes to improve the reproducibility of the work that we publish. This form provides structure for consistency and transparency in reporting. For further information on Nature Research policies, seeAuthors & Referees and theEditorial Policy Checklist .

Statistics

For all statistical analyses, confirm that the following items are present in the figure legend, table legend, main text, or Methods section.

n/a	Confirmed	
☒	☐	The exact sample size (n) for each experimental group/condition, given as a discrete number and unit of measurement
☒	☐	A statement on whether measurements were taken from distinct samples or whether the same sample was measured repeatedly
☐	☒	The statistical test(s) used AND whether they are one- or two-sided<br>*Only common tests should be described solely by name; describe more complex techniques in the Methods section.*
☐	☒	A description of all covariates tested
☒	☐	A description of any assumptions or corrections, such as tests of normality and adjustment for multiple comparisons
☒	☐	A full description of the statistical parameters including central tendency (e.g. means) or other basic estimates (e.g. regression coefficient) AND variation (e.g. standard deviation) or associated estimates of uncertainty (e.g. confidence intervals)
☒	☐	For null hypothesis testing, the test statistic (e.g. F, t, r) with confidence intervals, effect sizes, degrees of freedom and P value noted<br>*Give P values as exact values whenever suitable.*
☒	☐	For Bayesian analysis, information on the choice of priors and Markov chain Monte Carlo settings
☒	☐	For hierarchical and complex designs, identification of the appropriate level for tests and full reporting of outcomes
☒	☐	Estimates of effect sizes (e.g. Cohen's d, Pearson's r), indicating how they were calculated

Our web collection on statistics for biologists contains articles on many of the points above.

Software and code

Policy information about availability of computer code

Data collection	Amira v.5 (FEI, Hillsboro, Oregon), ImageJ 1.51h (NIH), Avizo Lite 9.3.0
Data analysis	PCA geomorph v3.1.3. Rv3.6.1

For manuscripts utilizing custom algorithms or software that are central to the research but not yet described in published literature, software must be made available to editors/reviewers. We strongly encourage code deposition in a community repository (e.g. GitHub). See the Nature Research guidelines for submitting code & software for further information.

Data

Policy information about availability of data

All manuscripts must include a data availability statement. This statement should provide the following information, where applicable:

- Accession codes, unique identifiers, or web links for publicly available datasets
- A list of figures that have associated raw data
- A description of any restrictions on data availability

The fossil materials C2019 2A233 are deposited in the collections of the Chuxiong Prefectural Museum, Chuxiong, Yunnan Province, China, CVP 148-6 is deposited at the Field Museum, Chicago, and YMP 4677 is deposited at the Yale Peabody Museum, New Haven, Conn. USA. The extant crocodilian material used in this study (ROM R7964) is part of the Vertebrate Paleontological Collections of the Royal Ontario Museum, Toronto, Ontario, Canada. All raw CT data are available upon request at the respective museums. The morphometric data are available from the corresponding author upon reasonable request.

Field-specific reporting

Please select the one below that is the best fit for your research. If you are not sure, read the appropriate sections before making your selection.

Life sciences study design

All studies must disclose on these points even when the disclosure is negative.

Sample size	Individual teeth from fossils
Data exclusions	Selection was made on the basis of availability
Replication	No experimental findings are reported
Randomization	Individual teeth from fossils were selected based on availability
Blinding	Not possible to be blinded

Reporting for specific materials, systems and methods

We require information from authors about some types of materials, experimental systems and methods used in many studies. Here, indicate whether each material, system or method listed is relevant to your study. If you are not sure if a list item applies to your research, read the appropriate section before selecting a response.

Materials & experimental systems

n/a	Involved in the study	
☒	☐	Antibodies
☒	☐	Eukaryotic cell lines
☐	☒	Palaeontology
☒	☐	Animals and other organisms
☒	☐	Human research participants
☒	☐	Clinical data

Methods

n/a	Involved in the study	
☒	☐	ChIP-seq
☒	☐	Flow cytometry
☒	☐	MRI-based neuroimaging

Palaeontology

Specimen provenance	Data collected from published literature or direct measurements of specimens located in natural history museums
Specimen deposition	Specimen data were collected from published literature. Original data based on specimens located in various recognized Natural History Museums, like the Field Museum, Chicago, USA and Chuxiong Prefectural Museum, Yunnan Province, China
Dating methods	No new dating was performed in this study.

☒ Tick this box to confirm that the raw and calibrated dates are available in the paper or in Supplementary Information.
