# Supplementary material for: Early Jurassic dinosaur fetal dental development and its significance for the evolution of sauropod dentition
Source: Nat Commun. 2020 May 7;11:2240. doi: 10.1038/s41467-020-16045-7 (PMC7206009; doi:10.1038/s41467-020-16045-7)
Supplement: Supplementary file 3 — Description of Additional Supplementary Files [file 41467_2020_16045_MOESM3_ESM.pdf]

Description of Additional Supplementary Files

File Name: Supplementary Movie 1
Description: Embryonic cranial material of the sauropodomorph dinosaur Lufengosaurus (Chuxiong Prefectural Museum C2019 2A233) with the dentition shown in red.
